# Maternal Impact on Serum Immunoglobulin and Total Protein Concentration in Dairy Calves

**DOI:** 10.3390/ani12060755

**Published:** 2022-03-17

**Authors:** Manuela Immler, Kathrin Büttner, Tanja Gärtner, Axel Wehrend, Karsten Donat

**Affiliations:** 1 Animal Health Service, Thuringian Animal Diseases Fund, Victor-Goerttler-Straße 4, 07745 Jena, Germany; manuela-immler@web.de (M.I.); tgaertner@thtsk.de (T.G.); 2 Unit for Biomathematics and Data Processing, Veterinary Faculty, Justus-Liebig-University Gießen, Frankfurter Str. 95, 35392 Giessen, Germany; kathrin.buettner@vetmed.uni-giessen.de; 3 Clinic for Obstetrics, Gynecology and Andrology with Veterinary Ambulance, Justus-Liebig-University Giessen, Frankfurter Str. 106, 35392 Giessen, Germany; axel.wehrend@vetmed.uni-giessen.de

**Keywords:** health monitoring, passive transfer of immunoglobulins, non-esterified fatty acids, birth monitoring

## Abstract

**Simple Summary:**

Ensuring adequate passive transfer of immunoglobulins is of particular importance for calf health status and longtime productivity. This observational field study focused on the influence of the prepartum cow’s health status on immunoglobulin transfer, with special emphasis on metabolic health, taking relevant management factors into account. Increased serum concentrations of non-esterified fatty acids in dams were positively associated with increased serum immunoglobulin concentrations in their calves. In addition to well-established management practices, such as feeding more than 2 L of colostrum at the first feeding of calves within four hours and with high-quality maternal colostrum, whole-day birth monitoring was positively related to immunoglobulin transfer. Our results provide evidence that, in addition to newborn management, prepartum energy metabolism in cows influences immunoglobulin transfer and also highlight the importance of health monitoring of close-up cows, as well as the importance of whole-day birth monitoring, to ensure calf health by timely colostrum supply and minimizing the risk of dystocia.

**Abstract:**

For dairy calves, sufficient supply with high-quality maternal colostrum is crucial to achieve adequate passive transfer of immunoglobulins. This observational cross-sectional study aimed to determine the influence of the prepartum metabolic status of dams on the serum immunoglobulin and total protein concentrations of their dairy calves, taking other relevant management factors into account. A total of 551 cows and their calves from 124 German dairy farms were included. Blood and urine samples of the cows were sampled 1 to 3 weeks before the expected calving date. Two generalized linear mixed effects regression models were fitted to the data. An increase in a dam’s prepartum serum non-esterified fatty acids concentration was associated with greater serum immunoglobulin concentration in her calf. Calves of herds with established birth monitoring at night showed greater serum immunoglobulin and total protein concentrations. Calves being fed more than 2 L of colostrum and higher Brix values for colostrum were related to greater serum immunoglobulin and total protein concentrations in calves. In conclusion, there is evidence that, besides timely and sufficient supply of high-quality colostrum to new-born calves, the prepartum metabolic status of cows and birth monitoring impact the passive transfer of immunoglobulins.

## 1. Introduction

Calves are born almost agammaglobulinemic due to the placental structure of the cow and depend on the absorption of immunoglobulins (Ig) after birth [1,2]. Providing newborn calves with high-quality maternal colostrum ensures passive transfer of immunity and is essential for the good health and longtime productivity of a dairy cow. Insufficient intake of Ig within 24 h after birth risks failure of passive transfer of immunity (FPTI) and can cause long-term damage, such as vulnerability to diseases preweaning and postweaning [3,4], lower average daily gain (ADG) [2], higher morbidity and mortality rates [4,5,6], reduced first and second lactation milk production [4,6,7], early culling [6] and economic losses [5,8].

Calves’ intestinal epithelial cells are capable of absorbing macromolecules, including Ig, non-selectively by pinocytosis for only 24 to 30 h after birth, depending on the time of first feeding [8,9,10]. After that initial time period, up to nine days of age [11], serum Ig or total protein (TP) concentration can be measured in serum either directly or indirectly by several methods to monitor transfer of Ig [12,13,14]. To achieve adequate passive transfer (APT), serum Ig concentrations ≥10 mg/mL, sampled between 24 to 48 h after birth, are to be aimed at [15]. Serum TP measurements are also commonly used for monitoring the passive transfer of Ig [4,6,16] and indirect proxy measurements, i.e., using refractometry, are easy-to-use on-farm tools for reliable estimation of serum Ig concentrations to monitor for FPTI [1,14,17]. Serum TP consists of the albumin fraction and the globulin fractions, with the main part being immunoglobulins, in particular IgG, when measured after colostrum intake. During colostrum intake, the albumin–globulin ratio changes in favor of the globulins [18]. Serum TP concentrations <4.0–5.5 mg/mL are the most common thresholds for FPTI [4,17].

Numerous factors related to feeding and rearing calves ensuring APT have been studied so far. As a priority, maternal colostrum of high quality, containing at least an Ig concentration of >50 mg/mL, is recommended [2,8]. Besides Ig, colostrum provides calves with proteins, fat, minerals and vitamins, growth factors, growth hormones and immune cells [19,20]. Due to the rapid decrease in the absorption rate of Ig, calves are supposed to be fed within the first 6 h of life at least 4 L or 8.5–10.0% of their body weight of colostrum [8,15,21]. The frequency with which calves are removed from the calving pen and the method of first colostrum administration influence the adequate supply of Ig. Removing calves less than twice a day from the calving pen and allowing calves to be nursed by their dams showed higher risks of FPTI compared with the use of nipple bottles or esophageal tubes [4,22,23].

In order to investigate the maternal impact on the calves’ metabolism and immune status, studies were conducted to examine the effect of energy contents in the dry period diet, fat supplementations, and environmental influences, such as heat stress. Calves from cows fed high-energy diets and additional fat showed improved immune response as well as better apparent efficiency of Ig absorption (AEA) from the small intestine and ADG [24,25,26]. Tao and Dahl [27] showed a negative effect of late-gestation heat stress and other factors on placental development. These factors result in fetal hypoxia and malnutrition and also reduce AEA and circulating Ig. Furthermore, the metabolic status of the dam during late gestation impacts colostrum quality as measured by Brix refractometry [28].

To the best of our knowledge, there are no studies available investigating the influence of the prepartum metabolic situation of the dam on the serum Ig and TP concentrations of the calf. The aim of this observational cross-sectional study was to investigate the association between the metabolic status of a dam and the transfer of Ig to her calf, taking into consideration relevant well-known management factors influencing passive transfer of Ig. We hypothesize that one or more metabolic parameters measured 1 to 3 weeks ante partum are related to serum Ig and TP concentrations in the calf.

## 2. Materials and Methods

### 2.1. Animals and Study Design

This observational cross-sectional study is part of the study whose design was published in Immler et al. [28]. The study was performed within the framework of the cattle health monitoring project of the Thuringian Animal Health Service, Germany. The project was approved by the Thuringian State Office for Consumer Protection, which is a competent authority for research ethics approval in Thuringia. They granted a formal waiver of the need for animal-use approval because the study was part of the official Cattle Health Monitoring Program in Thuringian Cattle Herds (2684-04-5-TSK-19-103). Every effort was made to minimize suffering during the sampling of blood and urine and during the clinical examination.

During December 2016 and January 2017, farmers from among the Thuringian Animal Health Service’s clientele and two large animal practices in Southern Bavaria, Germany were invited to take part in the study. Ultimately, 124 dairy farms voluntarily participated in the study. The mean herd size was 447 cows (min: 24, max: 1700); cows on all farms were housed in freestalls. Calves were housed in separate boxes. Cows of 96 herds were purebred German Holstein, and the other 28 dairy farms reared other breeds, including Simmental and Brown Swiss cows. Data and sample collection occurred from March 2017 to March 2018.

On each farm, 7 to 10 multiparous cows about 1 to 3 weeks prepartum were selected based on the expected calving date. When necessary, the farms were visited repeatedly to achieve the required number of animals. Overall, *n* = 1053 cows and their calves were involved. Cows clinically diseased (e.g., pneumonia, mastitis, fever) were not included in the study, except for lame and primiparous cows. Out of the 1053 originally tested cows, the following numbers of animals were excluded for data analysis: 85 (8.1%) parturitions with calving within 24 h, 21 (2.0%) cows with twin births and 12 (1.1%) cows with first parity. Fifty-eight (5.5%) cows were excluded due to missing colostrum samples and four (0.4%) cows were excluded due to missing values of urine creatinine concentration. Furthermore, 150 (14.2%) calves were excluded due to sampling within 24 h or after 9 days of age, 173 (16.4%) calves because they were not fed with colostrum from their own dam and 39 (3.7%) animals were excluded due to missing data regarding newborn management. Finally, *n* = 511 cows and their calves were included in the data analysis. In addition, the following data on newborn calf management was recorded for each farm on the basis of common practice assessment: time and amount of first colostrum feeding, colostrum quality testing, separate primary calf caretaker and birth control (Table 1).

### 2.2. Clinical Examination, Sampling Protocol

The clinical examination of the dam was described in detail by Immler et al. [28] and included rectal temperature, body condition score (BCS), lameness score and rumen fill. Any kind of vaccination of the cow during the actual dry period was queried. Four well-experienced veterinary specialists in cattle medicine performed the clinical examination. Scoring was standardized according to a specific training scheme in order to minimize interobserver variability. Following the clinical examination, blood was harvested from the coccygeal vein into a 10 mL sterile BD Vacutainer^®^ (Clot Activator Tube, Becton Dickinson, Plymouth, UK) with an 18-gauge needle, and urine samples were collected in tubes using a sterile stainless-steel bladder catheter.

Calves were sampled between 1 and 9 days of age. Blood was harvested from the jugular vein into a 10 mL sterile BD Vacutainer^®^ (Clot Activator Tube, Becton Dickinson, Plymouth, UK) with an 18-gauge needle.

Samples were transported to the laboratory within three hours after collection or were shipped on ice to the laboratory overnight. The blood samples were centrifuged at room temperature within 24 h after collection (30 min, 4800× *g*) and serum and urine samples were frozen at −20 °C until further processing. Colostrum samples were stored at 4 °C until analysis.

### 2.3. Laboratory Analysis

Samples were analyzed in the laboratory of the Thuringian Animal Health Service in Jena, Germany. The laboratory is an accredited veterinary test laboratory under license of the German Accreditation Body according to the quality standards of the German and European Standard DIN EN ISO 17025. Selected metabolites for cows were serum activities of creatine kinase (CK), glutamate dehydrogenase (GLDH) and aspartate aminotransferase (AST), as well as serum concentrations of TP, albumin, urea, cholesterol, non-esterified fatty acids (NEFA), bilirubin, calcium, magnesium, phosphorus and selenium. For urine samples, net acid base excretion was determined as well as concentrations of calcium, potassium, sodium and creatinine. Selected metabolites for calves were serum concentrations of albumin, TP and Ig.

Serum samples of cows were analyzed by automated spectrophotometry (Beckman Coulter Unicel DxC 600^®,^ Beckman Coulter GmbH, Krefeld, Germany) using the bromocresol green method for albumin (g/L), a timed endpoint method for inorganic phosphorus (mmol/L), cholesterol (mmol/L), total protein (g/L) and magnesium (mmol/L), an enzymatic determination method according to the International Federation for Clinical Chemistry for aspartate aminotransferase (nkat/L, enzyme code 2.6.1.1), glutamate dehydrogenase (nkat/L, enzyme code 1.4.1.2), urea (mmol/L) and creatine kinase (µkat/L, enzyme code 2.7.3.2), an indirect ion-selective potentiometry for calcium (mmol/L), the diazochloranilin method for bilirubin (µmol/L) and an enzymatic colorimetric method for non-esterified fatty acids (mmol/L). Serum samples of calves were analyzed for albumin (g/L) and TP (g/L), as described above for cows.

The serum Ig concentration of calves was measured photometrically using an indirect proxy measurement (precipitation with sodium sulfate solution) as follows: after pipetting 30 µL serum into each of two test tubes, 2000 µL of sodium sulfate solution was added into the test tubes and intensively mixed. Samples were incubated for 30 min in warm water (30 °C). Absorbance of samples was measured photometrically (photometer: PerkinElmer Lambda 11UV/VIS) at 530 nm against sodium sulfate solution, together with absorbances of control serum (Assayed Bovine Multi-Sera, Level 2, Randox Laboratories Ltd., Crumlin, UK) and standard solution. Immunoglobulin concentrations of the samples (g/L) were calculated according to the following formula:

cs = (As × cst)/st

where cs = Ig concentration of the sample in g/L, As = absorbance of sample; cst = Ig concentration of the standard solution in g/L, and Ast = absorbance of the standard solution.

Selenium serum concentration in each of the cow samples was analyzed using inductively coupled mass spectrometry (DIN EN ISO 17294: 2017-01) by an external laboratory (SGS Analytics Germany GmbH site Jena, former Synlab Analytics & Services Germany GmbH site Jena, Hamburg, Germany).

The urine samples of the cows were analyzed for sodium (mmol/L), calcium (mmol/L) and potassium (mmol/L) by indirect ion-selective potentiometry. Creatinine (mmol/L) was analyzed by the modified kinetic Jaffé method, and net acid–base excretion (mmol/L) was determined by the titrimetric method described by Kutas [29].

Colostrum samples were analyzed using the digital refractometer HI96811 (Hanna Instruments, Vöhringen, Germany) with a range of 0–50% Brix, according to the manufacturer’s instructions.

### 2.4. Statistical Analysis

Data were prepared using Microsoft^®^ Office Excel 2016 (Microsoft Corporation, Redmond, Washington, USA). All further computations were made with R version 4.0.4 (15 February 2021) (Free Software Foundation’s GNU project, 2018) using R-package lme4 and R-Function lmer.

Associations between the serum Ig concentration of calves and the continuous variables and categorial variables, as well as the associations between the serum TP concentrations of calves and the continuous variables and categorial variables, were calculated separately. A sample size calculation was not performed due to the observational character of this study and the wide range of parameters included in a multivariable approach. Pearson’s correlation coefficients were calculated to describe the associations between the variables in the respective models. For all fixed effects, correlation coefficients between −0.50 and 0.50 were calculated, except for ‘GLDH’ and ‘AST’ (*r* = 0.54) and ‘NABE’ and ‘potassium in urine’ (*r* = 0.59). It is assumed that correlation coefficients less than 0.7 do not point to collinearity among the independent variables. In addition, we checked this assumption by calculation variance inflation factors which ranged between 1.13 and 3.17. According to Hair et al. [30], only variance inflation values above 10 indicate multicollinearity. This was not the case in the present study. Generalized linear mixed effects regression models with hierarchically structured random effects (cow within herd) using the maximum likelihood method were fitted to the data to identify associations between the respective outcomes, Ig and TP concentration, cow level variables as predictors and ‘days before calving’ (DBC) as covariable.

For model building, stepwise backward elimination was carried out. In the first step, all possible independent variables and covariables available were included in the model. In the second step, only those variables were kept in the model with a *p*-value for the regression coefficient ≤0.5. In the third step, the reduced model from the second step was further modified as only those variables were kept in the model with a *p*-value for the regression coefficient ≤0.25. After these three backward elimination steps, the final model was created. Variables included in the initial model, with their medians, quartiles, minimums, and maximums (continuous variables) or frequencies (categorial variables), are given in Table 1 and Table 2. The covariable DBC and the following variables were not normally distributed and therefore log-transformed for analysis purposes: serum activities of CK, AST and GLDH, serum concentrations of cholesterol, bilirubin and NEFA, and urine concentrations of calcium and sodium. Breed, BCS, lameness, parity, vaccination, timing of first feeding, quantity at first feeding, colostrum quality testing, separate primary calf caretaker, birth monitoring and birth monitoring at night, as well as all metabolic variables, were included as fixed effects, and herd and cow within herd as random effects. The log-transformed variable DBC was included as covariable. The fit of the model was assessed using Schwarz’s Bayesian information criterion (BIC).

Variables included in the final model for serum Ig concentration of the calf are presented in Table 3. Variables included in the final model for serum TP concentration of the calf are presented in Table 4.

The Wald test was used to estimate the effect of BCS, breed, timing of first feeding, quantity at first feeding, colostrum quality testing, separate primary calf caretaker and birth monitoring at night on serum Ig and TP concentration. Furthermore, multiple comparisons were carried out for breed, quantity at first milking and birth monitoring at night. Statistical differences in least squares means were adjusted by the Bonferroni correction.

## 3. Results

Samples of all cows were collected, on average, 10.3 days before calving, ranging from 2 to 45 days, and calves were sampled, on average, 2.9 days after birth. The overall mean (+/− standard deviation) serum Ig concentration of the calves was 12.7 g/L (+/− 6.4), with a minimum of 2.0 g/L and a maximum of 36.8 g/L. The overall mean (+/− SD) serum TP concentration of the calves was 57.1 g/L (+/− 8.6), with a minimum of 36.9 g/L and a maximum of 94.8 g/L. For all metabolic variables, a detailed description is presented in Table 2.

### 3.1. Associations between the Serum Ig Concentrations of Calves and Cow-Level Variables

Higher serum Ig concentrations in calves were associated with increasing serum NEFA concentrations (*p* = 0.03) and Brix values (*p* < 0.01), and a negative association with calf serum albumin concentrations (*p* < 0.01) was identified. With respect to the Wald test, in herds with established frequent birth monitoring at night, serum Ig concentrations in calves were higher compared to herds with no birth monitoring at night (12.6 g/L vs. 10.5 g/L, LSM, respectively; *p* = 0.05). Calves fed more than 2 L of colostrum at first feeding had greater serum Ig concentrations compared to calves fed 1–2 L of colostrum (11.95 g/L vs. 9.69 g/L, LSM, respectively; *p* < 0.01; Table 5).

The associations between calf serum Ig concentration and BCS (*p* = 0.09), breed (*p* = 0.16), timing of first feeding (*p* = 0.14), dam’s serum albumin concentration (*p* = 0.25), log(bilirubin) (*p* = 0.19) and urea (*p* = 0.31) and creatinine levels (*p* = 0.24) were statistically not significant (Table 3).

### 3.2. Associations between Calf Serum TP Concentrations and Cow-Level Variables

With respect to the Wald test, higher serum TP concentrations in calves were related to established birth monitoring at night compared to no birth monitoring at night (55.5 g/L vs. 52.4 g/L, LSM, respectively; *p* = 0.03). Furthermore, calves fed more than 2 L at first feeding had higher serum TP concentrations compared to calves fed only 1–2 L (55.2 g/L vs. 52.1 g/L, LSM, respectively; *p* < 0.01). Regarding breed, calves of Holstein Friesian cows showed higher LSMs of TP concentrations (56.4 g/L) compared to Simmental (52.1 g/L, *p* = 0.05) and Brown Swiss cows (52.4 g/L, *p* = 0.04; Table 5).

A positive association with calf serum TP concentrations was identified for Brix values (*p* < 0.01). The associations between calf serum TP concentrations and ‘timing of first feeding’ (*p* = 0.15), separate primary calf caretaker (*p* = 0.09), DBC (*p* = 0.29), dam’s serum albumin concentration (*p* = 0.13), log(AST) (*p* = 0.25), log(bilirubin) (*p* = 0.30), calcium (*p* = 0.22), NEFA (*p* = 0.15), urea (*p* = 0.15), log(calcium in urine) (*p* = 0.21) as well as creatinine (*p* = 0.17) were statistically not significant (Table 4).

## 4. Discussion

High colostrum quality and its supply within 24 h postpartum are crucial to keep calves healthy [2,6,8]. Maternal factors influencing colostrum quality, as determined by Brix refractometry, were investigated recently [28]. Our study was performed to identify relationships between health and metabolic parameters of close-up cows and serum Ig and TP concentrations of their calves as measured between 1 and 9 days of age. Serum Ig and TP concentration of calves are two common parameters to indicate FPTI, if serum Ig concentrations below 10 g/L and serum TP concentrations below 4.0–5.5 g/dL are measured [4,6,31,32,33]. In this study, well-accepted variables influencing FPTI, such as colostrum quality and supply, were recorded as well so as to be able to account for them in the multivariable models and minimize confounding.

The most striking finding in this study was a positive relationship between the serum NEFA concentration of a dam and the serum Ig concentration of her calf. Metabolic changes during the transition period are challenging for dairy cows [34,35,36]. Lipolysis, and consequently elevated serum NEFA concentrations, indicate negative energy balances (NEB) during this period, ensuring the additional energy demand [35,36,37]. Various in vitro studies have substantiated the negative effects of serum NEFA concentrations in dams on peripheral blood mononuclear cell (PBMC) proliferation and reduced INF-gamma secretion [35,38]. In contrast, a positive association between serum NEFA concentrations and natural antibodies in milk was identified in dairy cows [39]. Recently, a link between prenatal immune stimulation of dams by repeated lipopolysaccharide challenge and serum NEFA concentrations of beef heifers at weaning was reported [40]. Similarly, our results suggest a biological link between an assumed immune challenge, raising serum NEFA concentrations in dams and increased serum Ig concentrations in calves. A possible explanation of this phenomenon might be a presumed association between increasing serum Ig concentrations in dams caused by immune stimulation and serum Ig concentrations in their calves [41], probably caused by increased colostrum Ig concentrations due to increased plasma Ig concentrations in dams. In general, there is evidence that the serum NEFA concentration of the dam, as an indicator of NEB, is involved in the function of the immune system. Predominantly, PBMCs and cytokine secretion are negatively influenced by raising serum NEFA concentrations in the dam [35,38,42]. A positive effect of serum NEFA concentrations in dams on serum Ig concentrations in calves has not yet been described. Further studies should take up these findings to clarify the biological background of this association.

Although BCS barely missed significance in our study, we observed that calves of thin cows (BCS < 3) showed higher serum Ig concentrations compared to the reference group of cows (BCS 3–4). This is in line with the results of another study on the relationship between BCS and lymphocyte function in periparturient cows, where overconditioned cows had lower IgM secretion and INF-gamma secretion compared to medium and thin cows [34]. Remarkably, a study on the presence of lymphocytes in colostrum found that BCS was positively related to the presence of lymphocytes in colostrum [43]. Taken together, there is some evidence that the body condition of dams is associated with immune function (PBMC function, cytokine secretion) and serum Ig concentration in calves. To the best of our knowledge, relationships between BCS and serum Ig concentrations in calves have not yet been studied; further investigations should clarify this issue.

Birth monitoring at night was associated with serum Ig and TP concentrations in calves. We assume a close link between whole-time birth monitoring and early supply of colostrum. Furthermore, care intensity might play a vital role in avoiding dystocia, which can cause acidosis, hypoxia and reduced suckling behavior in newborn calves. Moreover, pain reduces colostrum intake as well as APT [3,44,45,46]. Whole-day frequent birth monitoring, especially during the night, can avoid these weaknesses and can be recommended to improve colostrum supply and reduce the risk of non-vital calves.

A strength of our study is that we recorded variables reflecting colostrum supply to newborn calves and its quality and included them in our model, accounting for them in the multivariable models to avoid confounding. The management factors ‘volume of colostrum at first feeding’ and ‘timing to first colostrum feeding’ are well-known to be crucial factors in supplying calves with sufficient Ig [2] and therefore have been included in our model. Our results show that calves fed more than 2 L of colostrum at first feeding had higher serum Ig and TP concentrations compared to calves only fed 1–2 L. These results are consistent with those of other studies [15,21]. Even though ‘timing to first feeding’ showed no significance in our study, other studies have demonstrated its importance [21,47], and it is recommended to feed calves within 1–2 h after birth [2]. Furthermore, serum albumin concentration of calves, representing the largest protein fraction at birth, was included in the model because it is related to serum Ig concentration [18,48]. During the first 30 days of life, albumin concentration changes, with an initial decrease, followed by a continuous increase. In contrast, serum Ig concentration increases after colostrum intake and decreases continuously until the development of the immune function of the calves [18]. Similar results were obtained by Bogin et al. [48]. The calculated ratio of albumin to globulin describes the relative distribution of these two protein fractions and changes accordingly during the first weeks of life. If serum TP concentration is used to control for APT, high serum albumin concentrations can mask Ig deficiency [49].

In our study, we analyzed the most common variables to measure FPTI in calf blood serum in parallel, i.e., the serum concentrations of Ig and TP, allowing us to identify factors which are associated with both outcomes. As proxy measurements, precipitation with sodium sulfate solution for Ig and automated spectrophotometry for TP are commonly used in the field [14]; we determined the associations between management practices variables and serum Ig, as well as TP concentrations in calves and cow-level variables, independently. For example, birth monitoring at night was related to both outcome variables which improved the evidence of this finding. NEFA was associated with serum concentrations of Ig but not TP. On the one hand, this may reflect a lower level of evidence, and suggests, on the other hand, that associations with FPTI were identified when serum Ig concentrations were measured rather than serum TP concentrations. Nonetheless, when on-farm refractometry is used, measuring TP is a valuable test for screening for FPT because the convenience of on-farm testing allows a greater number of samples than laboratory testing [17]. For further investigations on factors influencing FPTI, serum Ig measurement may be the preferable variable.

A limitation of our study is that we recorded variables related to the supply of colostrum and the management of newborns at the herd level but not at the individual level. The hierarchical model enabled us to consider these as herd level variables, but accuracy was limited compared to individual recording. Taking into account that accuracy of individual recording by farmers would have been limited by interobserver variability, a herd-level classification in at most three categories can be considered a good compromise between accuracy and recordability.

Furthermore, a meaningful determination of a discrete sample size was limited due to the observational character of this study and a wide range of parameters included in a multivariable approach. Therefore, a sample size calculation was not performed, limiting the external validity of our study. Metabolic imbalances of prepartum cows seem to impact adequate passive transfer of Ig but pathophysiological backgrounds need to be further investigated.

## 5. Conclusions

From our results we conclude that the metabolism of the cow is related to passive transfer of Ig. We found evidence that the serum NEFA concentration of the dam prepartum might be a suitable candidate parameter to investigate this association. Regarding the pathophysiological backgrounds, an interaction of body condition, fat mobilization and immune reaction prepartum is assumed to be of particular importance and needs to be further investigated. We identified clear relationships between gamma globulin concentration and colostrum quality, as determined by Brix refractometry and the management of colostrum supply. Whole-day birth monitoring, which enables timely colostrum supply and minimizes the risk of dystocia, was proven to be a relevant factor to improve APT and calf health.

## Figures and Tables

**Table 1:** Categorial variables with the respective categories and associated numbers of observations of 511 dairy cow–calf pairs included in the data analysis.

Category	Numbers of Observations
BCS	
BCS 3–4	380
BCS < 3	48
BCS > 4	83
Lameness score	
None	457
Low grade	22
Moderate and high grade	32
Breed	
German Holstein	406
Simmental	48
Brown Swiss	57
Parity	
Second	198
≥Third	313
Vaccination of the cow	
Yes	281
No	230
Timing of first colostrum feeding (h)	
<2	180
2 bis 4	298
>4	33
Quantity at first feeding (L)	
1 to 2	146
>2	365
Colostrum quality testing	
Colostrometer	97
Optical refractometer	45
Digital refractometer	22
None	347
Separate primary calf caretaker	
Yes	206
No	305
Birth monitoring	
Every 2 h	420
Occasionally	79
Rarely	12
Birth monitoring at night	
Yes	252
No	246
Only heifers	13

BCS: body condition score.

**Table 2:** Medians, quartiles, minimums and maximums of metabolic variables and Brix values of 511 German dairy cows and their calves ^1^.

	Median	1st Quartile	3rd Quartile	Minimum	Maximum
Brix (%)	23.0	19.5	26.0	7.2	39.9
Days before calving of sampling	9.0	5.0	13.0	2.0	45.0
Concentration in serum (calf)					
Immunoglobulin G (g/L)	12.1	7.9	16.5	2.0	36.8
Total protein (g/L)	56.2	50.9	62.3	36.9	94.8
Albumin (g/L)	26.8	25.6	28.0	17.6	35.8
Concentration in serum (cow)					
Total protein (g/L)	65.2	61.4	69.4	47.1	86.0
Albumin (g/L)	32.2	31.1	33.5	22.3	37.2
Inorganic phosphorus (mmol/L)	2.02	1.83	2.22	0.87	3.23
AST ^2^ (nkat/L)	1057.0	926.0	1238.0	626.0	3520.0
Bilirubin (µmol/L)	2.83	2.30	3.44	0.76	13.94
Calcium (mmol/L)	2.36	2.29	2.44	1.61	2.89
Cholesterol (mmol/L)	2.24	1.88	2.67	0.56	6.14
CK ^3^ (µkat/L)	1.83	1.33	2.94	0.33	142.0
NEFA ^4^ (mmol/L)	0.26	0.23	0.33	0.17	1.61
GLDH ^5^ (nkat/L)	178.8	127.6	279.2	51.6	4995.0
Urea (mmol/L)	4.3	3.6	5.0	0.9	10.1
Magnesium (mmol/L)	0.93	0.86	0.99	0.63	1.38
Selenium (µmol/L)	0.68	0.57	0.77	0.10	1.05
Concentration in urine (cow)					
Calcium (mmol/L)	1.4	0.5	4.2	0.4	22.0
Potassium (mmol/L)	248.2	193.1	290.2	28.8	469.4
Creatinine (mmol/L)	11.9	8.6	15.8	1.5	36.5
Sodium (mmol/L)	33.1	5.5	78.0	5.0	287.4
Net acid-base excretion (mmol/L)	97.0	55.0	150.0	−60.0	340.0

^1^ Brix values measured in colostrum samples between March 2017 and March 2018 by digital refractometry. Metabolic variables measured in blood and urine samples collected during the close-up period for cows and after birth for calves. ^2^ AST: aspartate aminotransferase. ^3^ CK: creatine kinase. ^4^ NEFA: non-esterified fatty acids. ^5^ GLDH: glutamate dehydrogenase.

**Table 3:** Coefficients, SE and 95% CI of variables included in the final linear regression model regarding the association between serum immunoglobulin concentration of 511 calves between March 2017 and March 2018 as measured by an indirect proxy measurement and cow-level variables measured 1 to 3 weeks before calving, including Brix values of colostrum samples.

			CI 95%				
Item	Coefficient	SE	Lower Bound	Upper Bound	*p*-Value	Wald Test	*p*-Value
BCS						4.845	0.09
BCS 3–4	Reference	Reference			Reference		
BCS < 3	1.998	0.91	0.20	3.79	0.03		
BCS > 4	0.269	0.77	−1.24	1.78	0.73		
Breed						3.639	0.16
German Holstein	Reference	Reference			Reference		
Simmental	−1.510	1.22	−3.91	0.89	0.22		
Brown swiss	−1.891	1.05	−3.96	0.18	0.07		
Timing of first feeding (h)						4.002	0.14
<2	Reference	Reference			Reference		
2–4	−0.842	0.76	−2.33	0.65	0.27		
>4	−2.783	1.42	−5.59	0.02	0.05		
Quantity at first feeding (L)						8.305	<0.01
1–2	Reference	Reference			Reference		
>2	2.256	0.78	0.72	3.80	<0.01		
Birth monitoring at night						8.670	0.01
Yes	Reference	Reference			Reference		
No	−2.074	0.78	−3.62	−0.53	<0.01		
Only Heifers	−3.165	2.09	−7.28	0.95	0.13		
Brix value of colostrum (%)	0.213	0.05	0.12	0.32	<0.01		
Serum concentration of calves							
Albumin (g/L)	−0.786	0.14	−1.05	−0.52	<0.01		
Serum concentration of cows							
Albumin (g/L)	0.165	0.14	−0.12	0.45	0.25		
Billirubin (log) (µmol/L)	−2.341	1.76	−5.81	1.13	0.19		
NEFA ^1^ (log) (mmol/L)	4.939	2.27	0.48	9.40	0.03		
Urine concentration of cows							
Urea (mmol/L)	−0.240	0.24	−0.71	0.23	0.31		
Creatinine (mmol/L)	−0.060	0.05	−0.16	0.04	0.24		

^1^ NEFA: non-esterified fatty acids. BCS: body condition score, SE: Standard error. CI: Confidence interval.

**Table 4:** Coefficients, SE and 95% CI of variables included in the final linear regression model regarding the association between serum total protein concentration of 511 calves between March 2017 and March 2018 and cow-level variables measured 1 to 3 weeks before calving, including Brix values of colostrum samples.

			CI 95%				
Item	Coefficient	SE	Lower Bound	Upper Bound	*p*-Value	Wald Test	*p*-Value
Breed						9.863	<0.01
German Holstein	Reference	Reference			Reference		
Simmental	−4.288	1.66	−7.56	−1.01	0.01		
Brown swiss	−3.940	1.48	−6.86	−1.02	<0.01		
Timing of first feeding (h)						3.763	0.15
<2	Reference	Reference			Reference		
2–4	−0.865	1.03	−2.89	1.16	0.40		
>4	−3.755	1.94	−7.58	0.07	0.05		
Quantity at first feeding (L)						8.411	<0.01
1–2	Reference	Reference			Reference		
>2	3.072	1.06	0.99	5.16	<0.01		
Separate primary calf caretaker						2.907	0.09
Yes	Reference	Reference			Reference		
No	−1.512	0.89	−3.26	0.23	0.09		
Birth monitoring at night						8.565	0.01
Yes	Reference	Reference			Reference		
No	−3.082	1.08	−5.21	−0.96	<0.01		
Only Heifers	−2.427	2.85	−8.03	3.17	0.39		
Days before calving of sampling (log)	−1.432	1.43	−4.09	1.22	0.29		
Brix of the colostrum (%)	0.259	0.08	0.12	0.41	<0.01		
Serum concentration of cows							
Albumin (g/L)	0.331	0.22	−0.10	0.76	0.13		
ASAT ^1^ (log) (nkat/L)	−4.070	3.55	−11.05	2.91	0.25		
Bilirubin (log) (µmol/L)	−2.560	2.49	−7.46	2.34	0.30		
Calcium (mmol/L)	−3.850	3.12	−9.99	2.29	0.22		
NEFA ^2^ (log) (mmol/L)	4.882	3.42	−1.85	11.61	0.15		
Urea (mmol/L)	−0.504	3.42	−1.16	0.15	0.15		
Urine Concentration of cows							
Calcium (log) (mmol/L)	−1.162	0.92	−2.98	0.66	0.21		
Creatinine (mmol/L)	−0.099	0.07	−0.24	0.04	0.17		

^1^ ASAT: aspartate aminotransferase. ^2^ Non-esterified fatty acids. SE: Standard error. CI: Confidence interval.

**Table 5:** Least squares means (LSM) (SE), minimum and maximum of immunoglobulin and total protein concentration from the final model, measured in serum of 511 calves between March 2017 and March 2018, regarding breed, quantity at first feeding of colostrum and birth monitoring at night.

	Immunoglobulin Concentration			
	*n*	LSM (SE)	Minimum	Maximum
Quantity at first feeding (L)				
1–2	146	9.69 (1.01) ^a^	2.8	28.7
>2	365	11.95 (1.02) ^b^	2.0	36.8
Birth monitoring at night				
Yes	252	12.6 (0.76) ^a^	2.0	36.8
No	246	10.5 (0.96) ^b^	2.3	36.2
Only heifers	13	9.4 (2.18) ^ab^	2.8	16.8
	Total protein concentration			
Breed				
German Holstein	406	56.4 (1.27) ^a^	39.3	94.8
Simmental	48	52.1 (1.81) ^ab^	36.9	71.0
Brown Swiss	57	52.4 (1.65) ^b^	38.2	74.5
Quantity at first feeding (L)				
1–2	146	52.1 (1.36) ^a^	36.9	80.0
>2	365	55.2 (1.37) ^b^	39.3	94.8
Birth monitoring at night				
Yes	252	55.5 (1.01) ^a^	36.9	90.0
No	246	52.4 (1.31) ^b^	39.3	94.8
Only heifers	13	53.0 (2.99) ^ab^	40.2	62.6

^a,b^ Values with different letters indicate differences within each category (*p* ≤ 0.05). LSM (SE): Least squares means (Standard error).

## Data Availability

Data is contained within the article (Table 1, Table 2, Table 3, Table 4 and Table 5). Details of the laboratory analysis are available on request.
